# Editorial: Highlights in Cardiac Rhythmology: 2021

**DOI:** 10.3389/fcvm.2022.866883

**Published:** 2022-03-11

**Authors:** Matteo Anselmino, Gaetano Maria De Ferrari

**Affiliations:** Division of Cardiology, Department of Medical Sciences, Città della Salute e della Scienza di Torino Hospital, University of Turin, Turin, Italy

**Keywords:** arrhythmias, electrophysiology, technologies, innovation, cardiac

The World Health Organization declared the outbreak of severe acute respiratory syndrome coronavirus 2 (COVID-19) a pandemic state on 11 March 2020, and, ever since, healthcare professionals have promptly invested all efforts into fighting against COVID-19 with the goal of saving the lives of patients, friends, and family members. Every day, physicians fight against diseases, from those limiting quality of life to those threatening survival. Taking the Hippocratic Oath, they respect scientific obligations, profess warmth and empathy, and take full responsibility for their patients' bettering. Nevertheless, physicians are not used to facing a long-lasting health crisis. Reactions to the unexpected scenario have been palpable. A small group of people are being squeezed by a gigantic enemy and simply remain petrified. A few, guided by feelings of inferiority towards the unprecedented situation, have shifted their energies toward personal or domestic matters, limiting professional duties. The majority, however, have “only” felt disoriented. There is a need for corporate guidance and a sense of the strength of a community moving together towards a common goal, favoring collaborations and team or network formation; these are crucial elements of great 2021's scientific production in all fields, Cardiac Rhythmology included (Figure 1).

**Figure 1:**
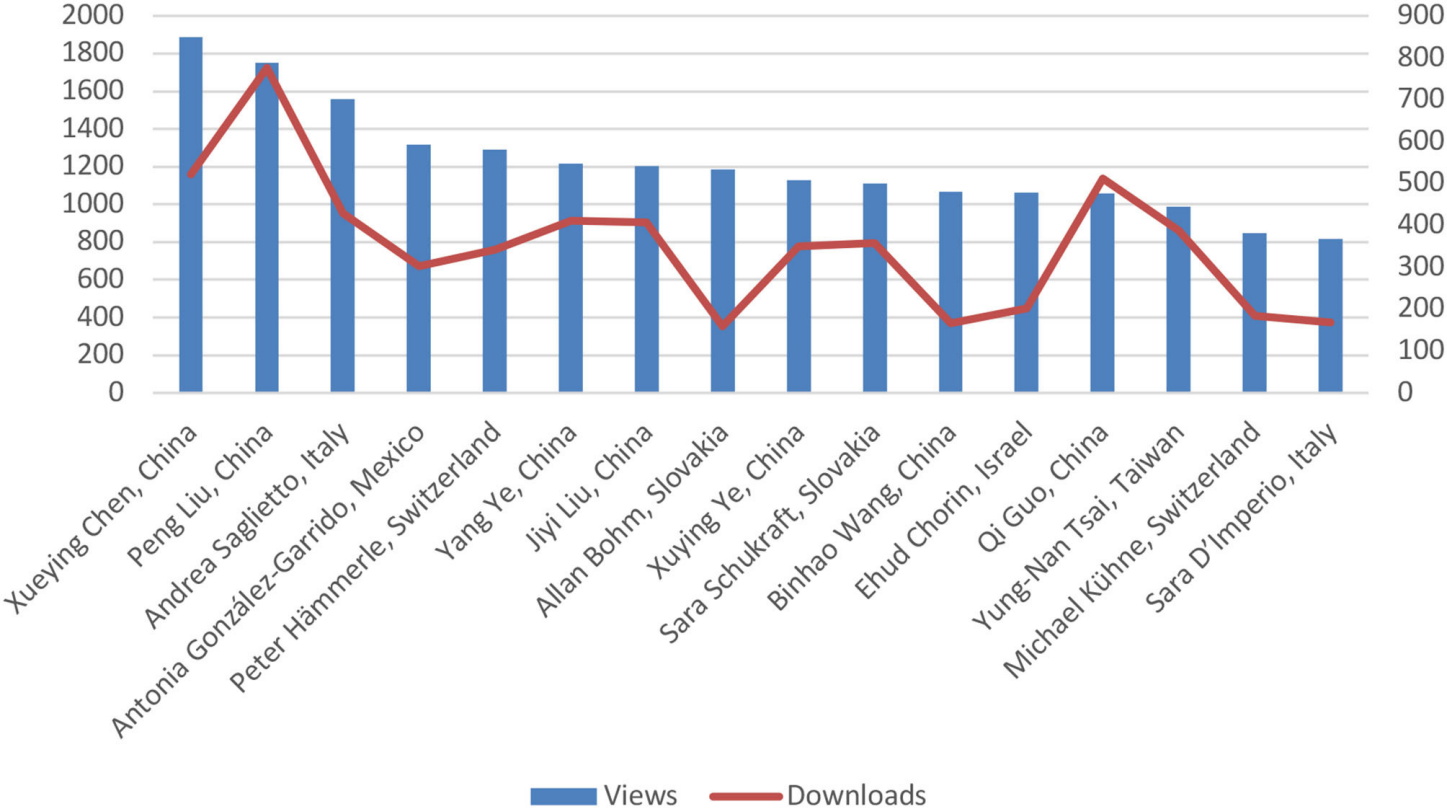
Metrics (at January 12th 2022) of the best-performing articles (First Author, Country) published in Frontiers in Cardiovascular Medicine, Rhythmology section, in 2021.

Out of the most creative and original topics, few emerge. Conduction system pacing (CSP), including left bundle branch pacing, is emerging as a promising pacing modality to prevent electrical and mechanical delay through direct capture of the original conduction system (Chen et al.; Liu P. et al.; Liu J. et al.; Ye, Wu et al.). The challenges related to the restricted number of tools initially confined CSP to small single-center experiences. As new tools are becoming available, the use of CSP is now spreading rapidly, even being used for distal conduction disturbances and, eventually, dealing with cardiac resynchronization. The clinical benefits of CSP are no longer in doubt, and apical pacing, particularly in patients with expected high pacing burden and initial structural heart disease, will soon be banded to avoid pacing-induced cardiomyopathy.

The same year a temporary, fully implantable pacemaker undergoing complete dissolution and clearance by natural biological processes was designed (1), the technology for continuous ECG monitoring and heart rhythm analysis by all kinds of wearable or miniaturized devices was validated [(2); Mancinetti et al.; Guo et al.]. Atrial fibrillation occupies a significant amount of attention due to the social and clinical burden of the arrhythmia. From thromboembolic risk markers, clinical management optimization, and new ablation sources and tools, innovations appear on a daily basis [Hämmerle et al.; Bohm et al.; Ye, Liu et al.; (3, 4)]. Early rhythm control, compared to usual care, has proved to decrease the risk of adverse cardiovascular outcomes (5) suggesting transcatheter ablation even as a first-line therapy option (Saglietto et al.).

Also, ventricular tachycardia management is experiencing a paradigm shift. Novel imaging protocols permit thorough tissue characterization and standardization of the origin depiction of arrhythmias. Insights into the candidate selection, safety, and efficacy of classical and innovative tools hold the potential to improve the outcome of this dreadful arrhythmia.

This issue includes a selection of the accomplishments of the Cardiac Rhythmology section from 2021; there is no time to rest—the wind blows strong in several directions. We do not yet know the future of parasternal access for substernal, less invasive, shock and pacing lead implantation, sympathetic nerve activity (measured at the skin or auditory canal levels), and alternative oxygen delivery and its impact on cardiac arrhythmias; however, we foresee good reasons to keep in touch also during 2022!

## Author Contributions

MA conceived the editorial. MA and GD revised the text. Both authors contributed to the article and approved the submitted version.

## Conflict of Interest

MA has received educational grants from Abbott, is consultant for Biosense Webster and proctor for Medtronic. The remaining authors declare that the research was conducted in the absence of any commercial or financial relationships that could be construed as a potential conflict of interest.

## Publisher's Note

All claims expressed in this article are solely those of the authors and do not necessarily represent those of their affiliated organizations, or those of the publisher, the editors and the reviewers. Any product that may be evaluated in this article, or claim that may be made by its manufacturer, is not guaranteed or endorsed by the publisher.
